# Current and Emerging Detoxification Therapies for Critical Care

**DOI:** 10.3390/ma3042483

**Published:** 2010-04-01

**Authors:** Brett A. Howell, Anuj Chauhan

**Affiliations:** Department of Chemical Engineering, University of Florida, Gainesville, FL, 32611, USA; E-Mail: bretthowell_28023@yahoo.com (B.A.H.)

**Keywords:** overdose, liposomes, emulsions, antibody fragments, fab, detoxification

## Abstract

Toxicity resulting from prescription drugs such as tricyclic antidepressants and cardioactive steroids, as well as drugs of abuse and exposure to environmental chemicals, represents a major need for detoxification treatments. Particles and colloids, antibody fragments (Fab), and indirect treatment methods such as macroemulsions, are currently being developed or employed as detoxification therapies. Colloids, particles, and protein fragments typically mitigate toxicity by binding to the toxin and reducing its concentration in vital organs. Indirect methods such as macroemulsions and sodium bicarbonate act directly on the affected organs, rather than the toxin. In this review, key design parameters (*i.e.* binding affinity, biocompatibility, pharmacokinetics) are discussed for each type of detoxification treatment. In addition, some of the latest research in each area is reviewed.

## 1. Introduction

Often times, prescription drugs designed to improve quality and/or length of life are accidentally or purposefully abused. This is also true of illicit drugs such as cocaine, heroin, and opioids. Such occurrences can lead to drug overdoses resulting in extreme discomfort, extended recovery times, and death. In addition, a significant number of people are exposed to toxins or poisons present in their surrounding environments, such as snake venom. Finally, new developments in chemical and biological warfare also bring possibilities of additional exposures to the forefront.

Many critical care treatments designed to indirectly reverse toxic modes induced by toxins have been used for several years in clinical practice. These include naloxone and sodium bicarbonate, among others. They are designed to treat the symptoms of overdose. Still, current treatments are not adequate as many patients continue to suffer and/or die from intoxication. The intravenous (IV) delivery of protein fragments, micro or nanoparticles, and colloids into a patient’s blood stream is a relatively recent and exciting platform for treating substance toxicity. Developments in this area have occurred over the past 30 years, with rapid improvements in the past 10–15 years due to significant advancements in the area of nanotechnology. Unlike treatments designed to treat the effects of toxicity, protein fragments and particles induce detoxification by redistributing it from the site of toxicity, which often includes the heart and/or brain, into the blood compartment. This results from specific or non-specific toxin binding. Below, the need for detoxification treatments in critical care situations is discussed, followed by a description of particles and colloids, protein fragments, and indirect treatment methods. A review of some of the most novel research taking place in each area is also presented.

## 2. The Motivation for Detoxification

Prescription drugs are a major target for many of the newest detoxification therapies being developed. Most of the adverse events associated with prescription drug deaths involve “poisoning,” which is defined as an overdose or the administration of the wrong drug [1]. A study conducted on the subject cited 55% more deaths where prescription drugs were listed as the underlying cause in 2003 *versus* 1999 (25,031 in 2003 compared to 16,135 in 1999) [1]. These figures show the magnitude of the prescription drug problem.

Many of the documented cases in [1] involved psychoactive, prescription drugs such as antidepressants. Tricyclic antidepressants (TCA) are one of the most life threatening types of antidepressants, with amitriptyline (AMI), dosulepin (DOS), and imipramine (IMI) being involved in many of the overdoses occurring [2]. In the United Kingdom, roughly 268 individuals die from TCA overdose each year [3], and the poison control centers in the United States report TCA poisoning as their third most reported type of poisoning [4]. Serotonin re-uptake inhibitors (SSRI) have become the new gold standard for the treatment of depression, but tricyclic antidepressants continue to be used for other symptoms such as migraine headaches, neuralgic pain, and attention deficit disorder [5,6], as well as depression where other medications are ineffective. Most overdose cases involving TCA’s are suicide attempts, although abuse for euphoria has also been documented [7].

TCA overdoses cause conduction disturbances in cardiac sodium channels [8], as well as interfere with processes more directly related to cardiac myocyte contraction and relaxation. They increase the open probability of ryanodine receptor (RyR) channels connecting the myocyte plasma membrane to the sarcoplasmic reticulum (SR) [9]. Ca^2+^ levels in the SR subsequently fall, reducing binding between actin and myosin filaments. AMI also inhibits sarco(endo)plasmic reticulum Ca^2+^ ATPase (SERCA) pumps from replenishing the SR with Ca^2+^ [9]. *In vitro* studies utilizing cardiac myocyte tissue have shown a concentration dependent decrease in contraction strength when exposed to AMI [10]. These effects lead to QRS interval elongation, oxygen deprivation owing to inadequate contraction, and cardiac arrest, which result in longer hospital stays for TCA’s *versus* other drugs [11]. TCA’s can have toxic effects on other body systems, such as the central nervous system (CNS), but cardiac disturbances are the primary concern.

As with TCA’s, local anesthetics can cause patient harm in the form of severe adverse reactions [12,13,14,15]. Bupivacaine (BUP) has the lowest toxic IV dose among the anesthetics [16]. As with TCA’s, BUP impairs cardiac contractility, possibly through direct Ca^2+^ related effects or interference with mitochondrial activity [16]. BUP can also disrupt the central nervous system (CNS) and cause seizures, delirium, and disorientation [16]. In addition, Na^+^ channels are again blocked in a concentration dependent manner [17]. Other drugs pose risks as well. Digoxin, a cardioactive steroid used to control heart rate and treat congestive heart failure, is marked by a narrow therapeutic index [18]. Thousands of toxic exposures and numerous deaths have been reported from cardioactive steroids [18]. They increase cardiac muscle contraction strength by inhibiting the Na^+^-K^+^-ATPase pumps and increasing the intracellular Ca^2+^ concentration. However, at elevated levels, this mechanism also leads to elevated potassium levels in the serum, conduction disturbances, and general cardiac conduction system dysfunction [18]. Fentanyl, an extremely potent opioid pain killer, also presents a danger to many patients [19,20].

Although prescription drugs are currently the most aggressively targeted cause of drug toxicity, drugs of abuse embody an even greater problem and could be another target for therapies in the future. Cocaine, heroin, morphine, and street derivatives thereof kill many people, especially in inner city areas [21]. Opioids primarily lead to respiratory depression, although serious cardiovascular effects can occur in select instances [22]. Cocaine abuse, often in combination with alcohol leading to the toxic metabolite cocaethylene [23,24], can result in myocardial ischemia or infarction [25].

Exposures to organophosphates, both in the context of warfare and agricultural treatments, make up a large number of poisonings world-wide. To date, major attacks with such chemicals have been limited. In 1995, sarin gas was purposely emitted into the subway system in Japan. This event serves as a reminder of the wide-spread fear and damage large scale attacks could impose. Effective, rapid treatments could mitigate some fears. Beyond the scope of warfare, organophosphates used as pesticides have resulted in a great number of poisonings in developing nations [26]. In addition, more obscure and potent toxins such as the venom of deadly snakes also take lives. Together, the aforementioned dangers have led to the emergence of several treatment options, but continue to provide researchers with the impetus to develop more robust ones as well.

## 3. Treatment Methods

The treatment methods used or being developed for critical care situations will be discussed here under three broad headings. First, colloids and particles (hereafter referred to as “vehicles”) such as liposomes, micro-emulsions, spherulites, and polymers will be considered, followed by drug specific antigen-binding fragments (Fab). Lastly, indirect treatment methods such as naloxone and sodium bicarbonate are covered.

### 3.1. Colloids and Particles

**Figure 1:**
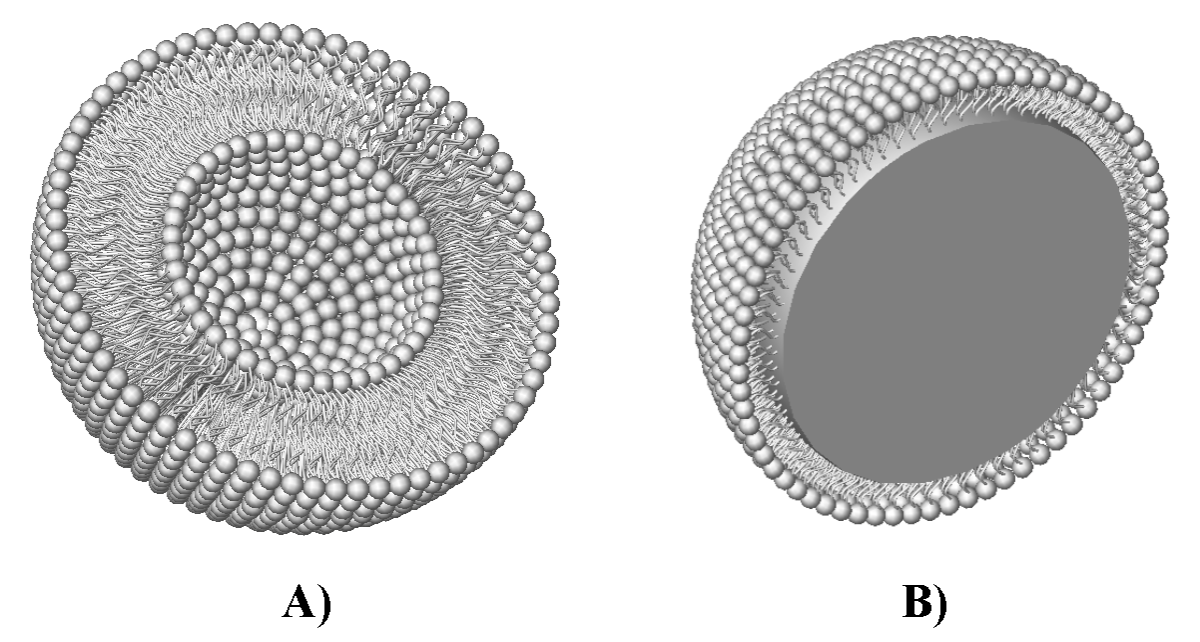
Colloids used for detoxification, including (a) unilamellar liposomes, comprised of a lipid bilayer composed of phospholipids surrounding an aqueous core, and (b) microemulsions or nanocapsules, which include an oily core stabilized by either phospholipids or surfactants.

The most heavily explored colloids for drug overdose treatment include liposomes and similar lipid-based colloids, as well as microemulsions. Liposomes are vesicles composed of phospholipids, usually suspended in an aqueous media. Phospholipid head groups comprise the inner and outer surfaces, while aliphatic tails make up the bilayer region (Figure 1a). Some common phospholipids used to make liposomes include phosphatidylethanolamine (PE), phosphatidylcholine (PC), phosphatidylserine (PS), and phosphatidylglycerol (PG), although many more have also been utilized. Varying the head groups and aliphatic tails allows changes in surface charge and bilayer properties [27]. Liposomes are made *via* energy intensive processes such as sonication or extrusion, or solvent evaporation methods [28,29]. Owing to their unique structures that include an inner aqueous compartment and a lipid bilayer, liposomes have a variety of uses besides detoxification. Many studies have focused on their drug delivery capabilities, with products such as DOXIL^®^ and CAELYX^®^ already on the market for the delivery of doxorubicin to tumors. Liposomes also serve as great mimics of the cell membrane, and can be used for *in vitro* to *in vivo* assays [30] and fundamental membrane studies. Spherulites are colloids similar to liposomes except they have extremely ordered bilayers [31]. The bilayer order can increase their entrapment efficiencies in some cases. Nanocapsules are also composed of phospholipids but have an oily core [32]. As oil-core colloids, they are similar in structure to microemulsions, but simultaneously possess some qualities of liposomes because of their compositions.

Microemulsions are oil droplets in aqueous phases or aqueous droplets in oil phases stabilized by surfactants. An illustration of the microemulsion structure is shown in Figure 1b. Additional surfactants such as fatty acids can be added to alter the properties of microemulsions [33]. As their name suggests, microemulsions are usually 0.5 to several microns in size. Unlike liposomes, microemulsions often form spontaneously at optimal oil to surfactant ratios, but may become unstable upon dilution. Block copolymer surfactants have recently become an interesting area of study for many researchers working in this field, as their structures may be easily tailored for a particular end use. In addition, polymeric particles have been investigated as drug sequestering agents. They are usually solid, porous hydrogels or cross-linked structures, and may be functionalized as well. At the present time, the colloids and particles under study would be almost exclusively dosed intravenously after toxicity, due to the complexities associated with crossing the gut lumen and the need for stable environments to maintain desired properties.

#### 3.1.1. Important Design Parameters

The ultimate goal for most detoxifying vehicles is drug redistribution. They are tailored to maximize drug-particle binding so that unbound drug molecules in the blood stream will be sequestered upon intravenous dosing. This sequestration then triggers drug molecules in the tissues to relocate to the blood compartment, thereby inducing a shift of drug molecules away from vital organs such as the heart and central nervous system. Accordingly, the binding characteristics between the vehicles and the targeted moieties are the primary concern. Modifications in surface properties, charge distribution, functional groups, and structures of the treatment vehicles can improve their affinity for the toxin. Much of this work can be done *in vitro* prior to animal studies. When carrying out the *in vitro* experiments, it is important to test binding in the presence of serum proteins and in some cases, blood cells, that will compete with the detoxifying vehicles for toxin binding *in vivo*. A small number of detoxification vehicles are designed to actively neutralize the toxin of interest, in which case the kinetics of binding also play a major role.

While vehicle-toxin binding affinity is the first property of interest when designing a redistributing system, the pharmacokinetic properties also matter a great deal. Pharmacokinetic properties of importance include the amount of time the vehicles remain in circulation in the venous and arterial blood, as well as the means by which they are removed from the blood. When foreign bodies like liposomes, microemulsions, and polyermic particles are introduced into the blood compartments, serum proteins bind to their surfaces [34]. This induces opsonization where the immune system coats the vehicles with recognizable antibodies so they can be targeted for destruction by various immune cells [35]. Not only does this process lead to the elimination of the detoxifying vehicles during their initial dose, but can also allow the body to store information about the vehicles for much faster recognition upon subsequent dosing [36]. Various vehicle properties like composition [37], surface charge [35,38], size [35,39], and surface modifications can transform their pharmacokinetic properties. The most widely used and successful surface modifications are polymer coatings such as poly(ethylene glycol) (PEG) to shield protein binding and delay opsonization. Most PEG layers are around 5 nm thick [40] and have been shown to increase circulation times [41] and reduce liver and spleen accumulation for liposomes [41,42]. Optimal coverages are roughly 5–10%, while too much polymer can disturb the structure of the particle or liposome and reduce binding [42,43,44].

A third and final parameter to consider when designing colloids and particles for detoxification is biocompatibility. The treatment method should have minimal side effects and never increase a patient’s risk level beyond the level prior to treatment. Short term side effects may be present in some cases, and must be balanced against the risk of withholding treatment. Liposomes have been used as drug delivery vehicles for several decades [45]. Since phospholipids are naturally present in the human body, they are usually non-toxic with a few exceptions. Microemulsions contain surfactants and large proportions of oil. Certain surfactants and oils are generally regarded as safe, while further testing is required for others. Polymeric particles can be cytotoxic, especially those that are cationic in nature. All possible modes of vehicle-induced toxicity must be examined during the *in vitro* development phase.

#### 3.1.2. Current and Emerging Colloid/Particle Therapies

**Figure 2:**
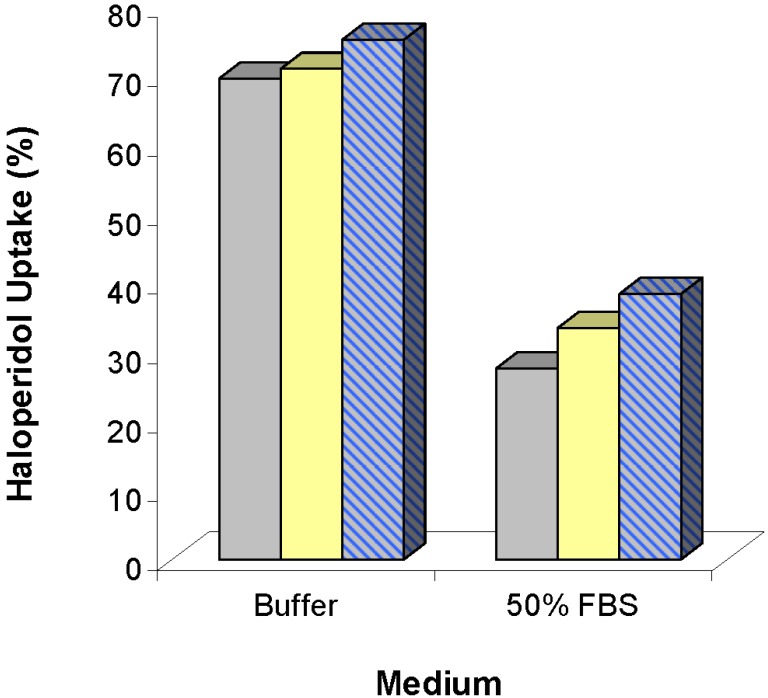
Haloperidol uptake by multilamellar (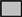) and unilamellar (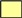) liposomes and spherulites (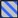) in the presence and absence of 50% fetal bovine serum (FBS), adapted from Dhanikula *et al.* [31] with permission.

Significant progress towards viable lipid-based detoxifying vehicles has been made by Dhanikula *et al*. Spherulites composed of cholesterol, neutral lipids, and pegylated lipids were designed to sequester the drugs haloperidol, docetaxel, and paclitaxel [31]. Internal aqueous compartments had pH values of 3 and contained albumin in some cases to enhance drug entrapment. Maximum drug binding of 75.2% for haloperidol, 94.4% for docetaxel, and 91.5% for paclitaxel was observed, although Figure 2 shows evidence for reduced binding in the presence of serum proteins.

**Figure 3:**
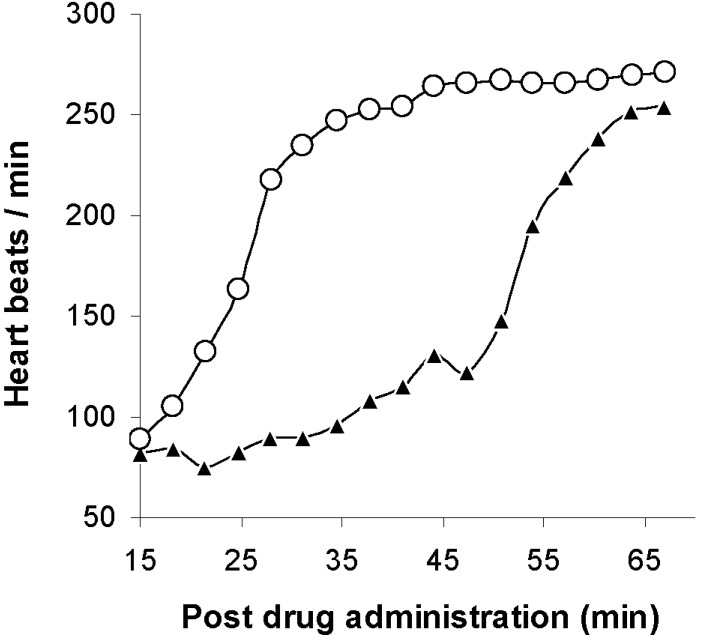
Heart rate recovery in rats treated 15 min after amitriptyline administration with spherulites (O) and no spherulites (▲), adapted from Dhanikula *et al.* with permission [46].

When the spherulites were tested for AMI binding, spherulites with internal pH gradients were capable of sequestering 98% of dissolved AMI in buffer and 97% in the presence of 3% albumin [46]. Following the *in vitro* studies with spherulites, they were injected into isolated rat hearts exposed to AMI [46]. Figure 3 clearly demonstrates a faster recovery for the treated *versus* the untreated rat hearts. The recovery was roughly twice as fast with treatment. Besides using spherulites for detoxification, Dhanikula *et al*. also assessed the drug uptake properties of nanocapsules with oily cores [47]. The best oils for binding were miglyol 810 and tricaprylin. Drug binding in aqueous buffer solutions did not exceed 75% for haloperidol, docetaxel, or paclitaxel. When measured in the presence of serum proteins, binding values fell even further, and ranged from 0% to 61%.

**Figure 4:**
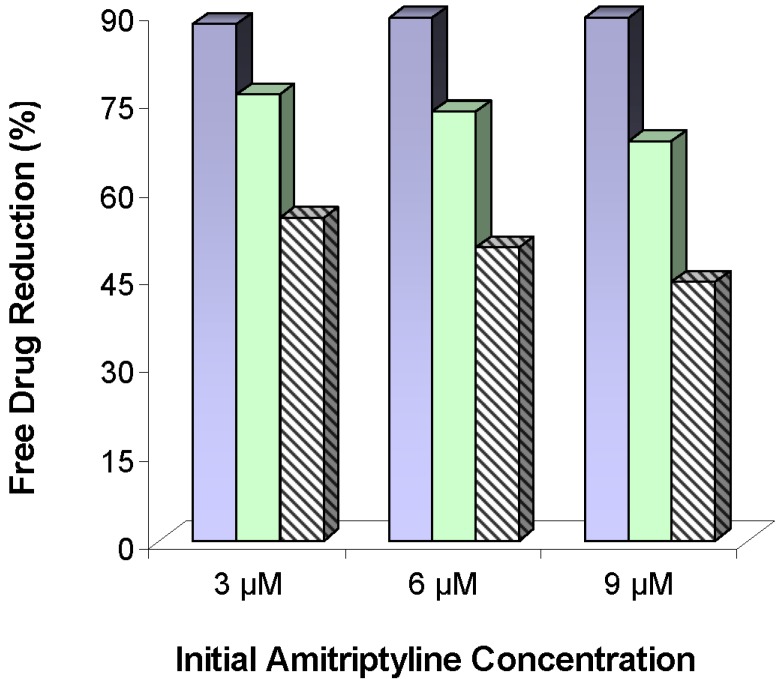
Free drug concentration reductions of amitriptyline in human serum by 95:5 DOPG:DPPE-mPEG-2000 liposomes at loadings of 1.44 (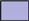) and 0.72 (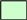) mg lipid/mL, and 50:50 DMPC:DOPG liposomes (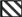) at 0.72 mg lipid/mL. Free drug reductions were calculated based on the differences between free drug concentrations in human serum samples and free drug concentrations in human serum samples mixed with liposomes. Figure adapted from [50].

Fallon and Chauhan investigated liposomes with various proportions of negatively charged lipids for their AMI binding capabilities [48]. Among other conclusions, they found that anionic liposomes were more effective at sequestering cationic drugs than neutral liposomes, and that drug binding was rapid and pH dependent. Howell and Chauhan continued their work by comparing the binding affinities of isolated serum proteins such as albumin, fibrinogen, and globulins, with liposomes partly or fully composed of the anionic phospholipid 1,2-Dioleoyl-*sn*-Glycero-3-[Phospho-rac-(1-glycerol)] (DOPG) [49]. DOPG liposomes showed high affinities for AMI, binding over 99% of the drug in buffer solutions. The presence of serum proteins interfered with the liposome-drug complexation, both with mixtures of isolated proteins and in human serum samples. The problem was remedied with the inclusion of phospholipids including a covalently attached PEG chain [50]. The PEG layer allowed for similar drug-liposome attractions in buffers but helped to shield serum proteins in human serum solutions. Figure 4 confirms the difference in the free drug reduction for liposomes composed of 50% anionic lipids and 50% net neutral lipids with no PEG chains *versus* anionic liposomes with PEG chains [50]. Although PEG chains were effective shields at 5% by mole inclusion, too much PEG reduced drug binding by disrupting the bilayer structure. After identifying pegylated, anionic liposomes ostensibly capable of *in vivo* efficacy, they were tested for packaging effects and showed no change in sequestering abilities after storage for 1 month.

**Figure 5:**
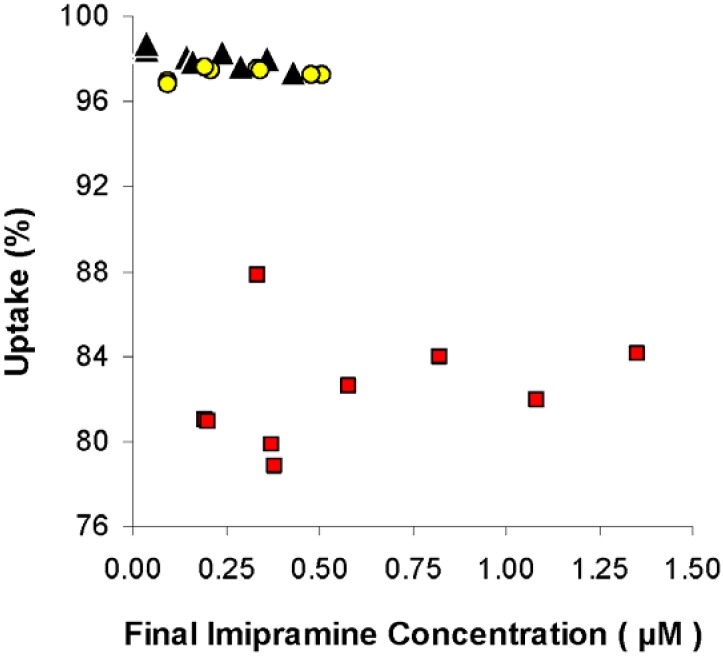
Percent imipramine uptake by human serum (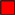) and by a mixture of human serum and 95:5 DOPG:DPPE-mPEG-2000 liposomes at 1.44 mg lipid/mL (▲) and a mixture of human serum and 95:5 DOPG:DPPE-mPEG-5000 liposomes at 1.68 mg lipid/mL (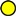) *versus* final imipramine concentration. Figure adapted from [44].

Following their work with AMI, Howell and Chauhan did further *in vitro* binding experiments with other TCA’s [44]. The same liposomes were capable of sequestering imipramine (IMI) and dosulepin (DOS) as effectively as AMI. Interestingly, binding of opipramol, a diprotic drug with only 83% of its population protonated *versus* 99% for the TCA’s, was less extensive. This again confirmed the importance of charge-charge interactions. The length of the PEG chain incorporated into the liposomes was also probed and shown to be inconsequential. Figure 5 illustrates high IMI binding for PEG-2000 and PEG-5000 chains [44].

**Figure 6:**
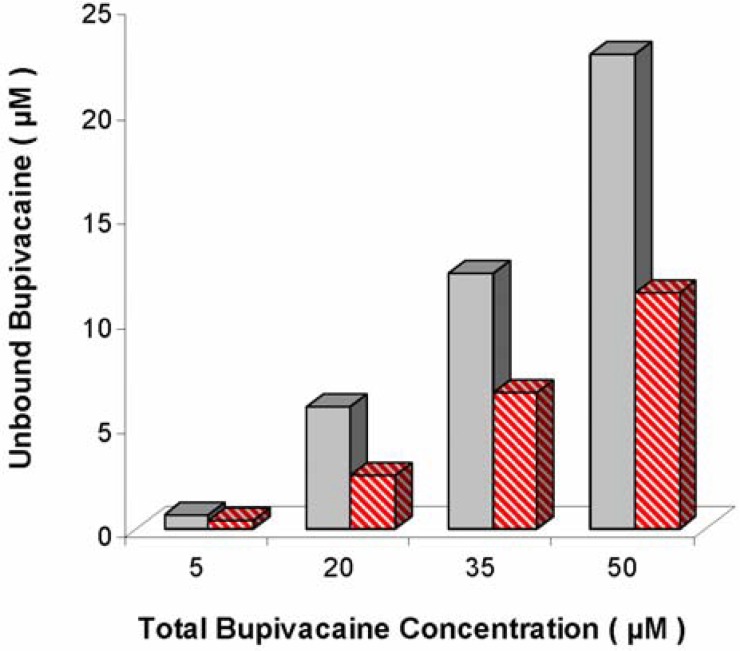
Free bupivacaine (unbound to proteins and/or liposomes) *versus* total bupivacaine concentration in human serum samples in the absence (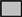) and presence (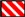) of unilamellar, 95:5 DOPG:DPPE-mPEG-2000 liposomes at 2.9 mg lipid/mL. Differences were significant at all concentrations tested (P = 0.037, 0.022, 0.042 and 0.018 for 5, 20, 35, and 50 µM, respectively). Figure adapted from [51].

In an attempt to develop a detoxification strategy aimed at multiple moieties, BUP binding with pegylated, anionic liposomes was also tested (Figure 6) [51]. Free BUP concentrations were significantly reduced with liposomes in human serum solutions.

**Figure 7:**
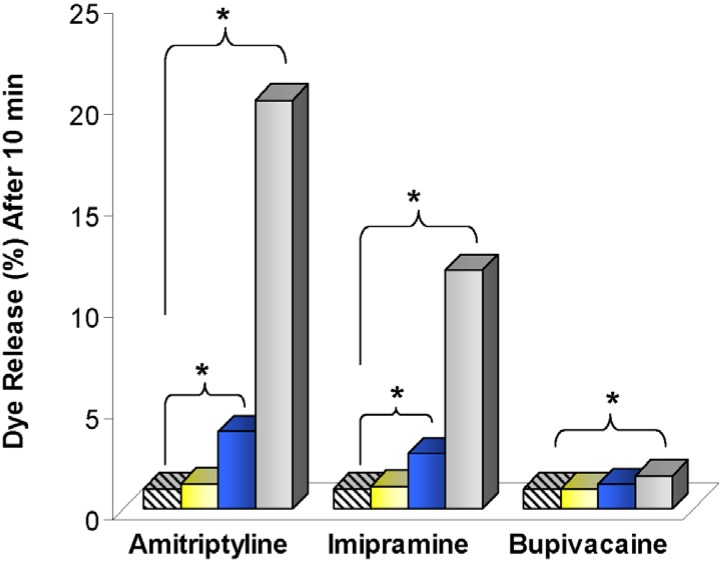
Percent of entrapped calcein released by 95:5 DOPG:DPPE-mPEG-2000 liposomes 10 minutes after exposure to amitriptyline, imipramine, or bupivacaine at drug concentrations of 0 (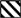), 8.6 (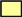), 38 (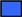), and 150 (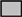) μM. Means are shown with n = 2-4. Marker denotes p < 0.05 (*). Figure adapted from [52].

Next, Howell and Chauhan sought to understand why liposomes and drugs were complexed so effectively, and how binding differed across drug types. Interaction studies were done by using solutions of high ionic strengths, various lipids, and various liposome structures [52]. While reducing the Debye lengths and shielding charge-charge interactions lessened liposome-drug binding, lipid bilayer properties also played a major role. When combined with liposome leakage results where a fluorescent dye was released from liposome cores upon drug exposure (Figure 7), the experiments led to several conclusions. Charge-charge interactions brought drugs into close proximity with liposomes, while lipophilic interactions allowed the drugs to be retained. Due to structural differences, TCA’s were capable of achieving a lower energy state within the lipid bilayers when compared to BUP, which led to increased dye leakage for the TCA’s. The experimental results were validated with double layer theory calculations and a Langmuir binding model.

**Figure 8:**
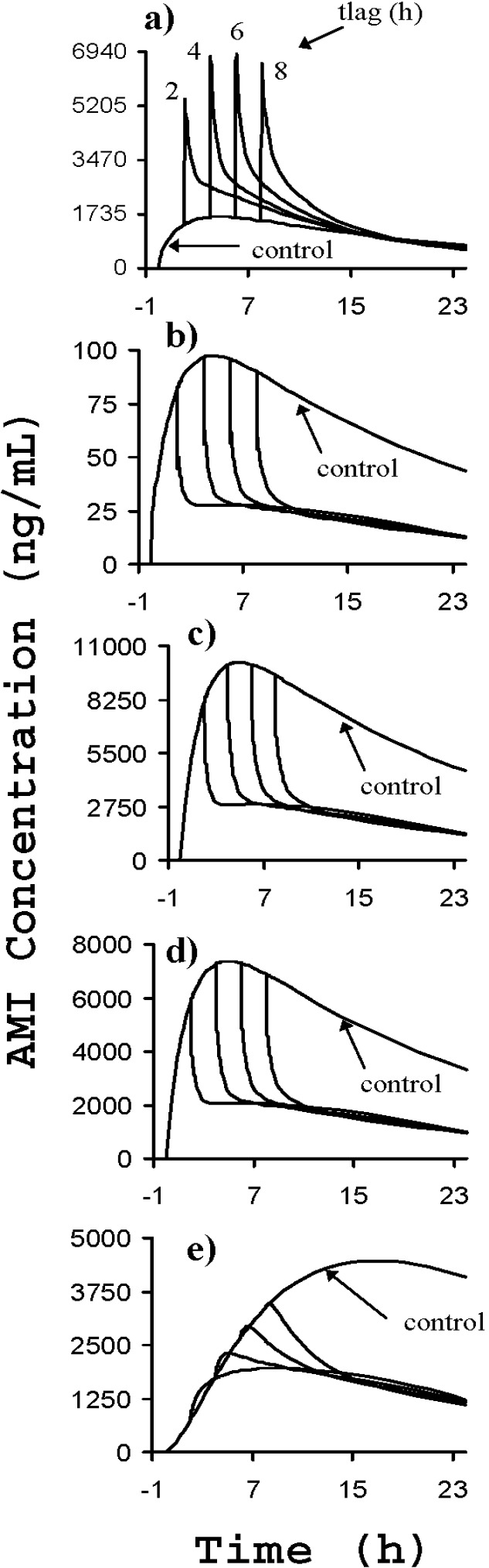
Amitriptyline concentrations *versus* time for simulated overdose cases (2500 mg) without liposomes and with liposomes at 1.44 g lipid/L for t_lag_ = 2, 4, 6, or 8 hours for a) venous whole blood, b) free AMI in venous blood, c) heart, d) brain, and e) muscle. T_lag_ refers to the time lapse between AMI ingestion and liposome treatment. Figure reprinted from [53].

Lastly, Howell and Chauhan approximated whether liposomes could compete with tissues and proteins *in vivo* with physiologically based pharmacokinetic (PBPK) models [53]. Organ to blood partition coefficients for TCA’s and BUP were obtained from published equations [54] and the *in vitro* data discussed above. Various overdose or adverse reaction scenarios were simulated after the models were validated with intravenous data. In Figure 8, the effect of introducing liposomes into the venous blood compartment 2, 4, 6, or 8 hours after initial AMI ingestion is displayed. The PBPK models predicted excellent redistribution for AMI, with area under the drug concentration *versus* time curve (AUC) reductions of over 60% for the heart and brain with liposomes, compared to the control case. BUP redistribution was also clear but less drastic (≈15%). The models provided some insight concerning the details of drug overdose treatment with liposomes under clinical circumstances. The liposomes may be effective up to 8 hours after AMI ingestion, optimal liposome doses were forecasted, and local pharmacodynamic improvements in the heart were predicted.

**Figure 9:**
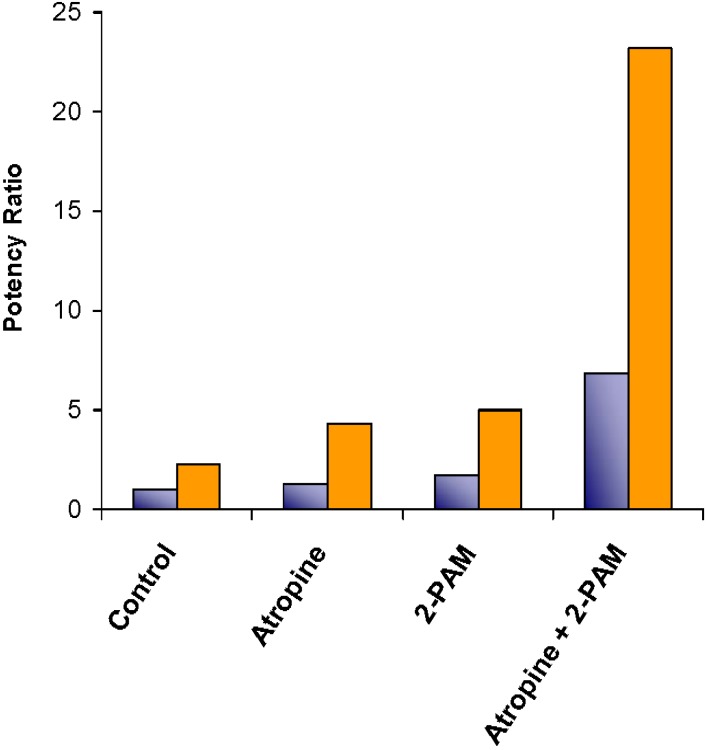
Potency ratios for the organophosphate antagonists atropine and 2-PAM without sterically stabilized liposomes (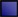) and with sterically stabilized liposomes (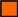) in mice. The potency ratio is the LD_50_ of the organophosphate diisopropylfluorophosphate (DFP) with the antagonist divided by the LD_50_ of DFP without the antagonists. Adapted from Petrikovics *et al.* with permission [58].

In addition to the lipid based colloidal research mentioned thus far, Petrikovics *et al*. have also done a considerable amount of work in this area. While they utilize liposomes for detoxification, the liposomes serve as drug delivery devices carrying enzymes throughout the body to impose active toxin breakdown, as opposed to redistribution. Most recently, they have studied liposomes carrying the enzyme rhodanese to aid in converting toxic cyanide to less toxic thiocyanate [55]. Various liposome formulations were tested for their relative rhodanese activity and 1-palmitoyl-2-oleoyl-*sn*-glycero-3-phosphocholine/cholesterol/1,2-dipalmitoyl-sn-glycero-3-phosphoethanolamine-N-methoxy(polyethylene glycol)-2000] (POPC/CHOL/PEG-PE-2000) liposomes at a molar ratio of 57/38/5 were shown most active. Organophosphorous intoxications have been targeted as well [56,57,58,59,60]. The organophosphorous hydrolyzing enzyme, organophosphorous acid anhydrolase (OPAA), was encapsulated in pegylated liposomes [58]. After reporting *in vitro* data confirming the enzyme activity, mice were treated with several different treatment methods and death was induced with diisopropylfluorophosphate and lethal doses were noted. Atropine and pralidoxime (2-PAM) were given separately as controls, and simultaneously with liposomes to compare treatment methods. As the results in Figure 9 show, liposome-encapsulated OPAA, in combination with traditional treatment methods, increased the lethal doses significantly.

Besides liposomes, microemulsions are another detoxification vehicle under study [33,61,62]. Varshney *et al.* experimented with a variety of surfactants as stabilizers for ethyl butyrate and found that Pluronic F-127 produced the highest binding affinities for BUP. The ethyl butyrate (1 mg oil/mL)/Pluronic F-127 combination extracted 60% of BUP from solutions [33]. Working with AMI, James-Smith *et al*. conducted a more detailed interactions study and suggested that 12 TCA molecules were binding to each oil droplet [62]. Charge-charge interactions were mentioned as important for drug-droplet attraction.

Nanocapsules stabilized by a different surfactant, Brij 97, and further altered with polysilicate/polysiloxane shells, were tested for their BUP binding affinities [63]. Both BUP and quinoline were studied. Greater than 99% of BUP was bound to the microemulsions, while 97% of quinoline was taken up. Drug binding was attributed to hydrophobic interactions between the lipophilic drugs and the oil phases. Alternatively, Chakraborty and Somasundaran used polymeric particles to remove AMI and BUP from saline solutions [64]. Their particles were made of poly(acrylic acid), and thus highly charged. At relatively high AMI concentrations, extraction efficiencies were 80% and 63% for AMI and BUP, respectively. The results correlate with the proportion of each drug in the protonated state at a pH of 7.4. Furthermore, Lee and Baney focused on using dinitrophenyl chitosans produced from chitosan polymer/dinitro-fluorobenzene reactions to bind AMI [65]. The chitosans took up 90% of AMI from saline solutions at polymer concentrations of 0.4%. To simultaneously study the interaction mechanism responsible for the drug-chitosan binding and assess the toxicity of the chitosans, Lee *et al.* did NMR experiments and *in vivo* studies with rats [66]. NMR results uncovering proton shifts pointed to aromatic-aromatic interactions between the altered chitosans and the drugs. Similarly to several papers discussed above, the authors ultimately attributed the binding affinity to a combination of electrostatic and hydrophobic interactions. The altered chitosans were non-toxic in rats.

### 3.2. Antibody Fragments (Fab)

Besides colloids and emulsions, natural binding moieties are also effective at detoxification. Antibody fragments in particular have been very effective for this purpose. When foreign bodies, which include viruses, bacteria, certain small molecules, and others, invade the human blood stream, the immune system recognizes those bodies and begins producing antibodies that can subsequently induce opsonization. Certain substances are naturally highly protein bound, and therefore bind with extremely high affinities to their substance-specific antibodies. It was this idea that first inspired researchers to take advantage of the body’s antibody producing abilities to treat digoxin toxicity [67]. The procedure for antibody isolation is fairly complex. Sheep are injected with the substance of interest. The immune systems of the sheep begin to produce substance-specific antibodies some time later. Proteins from the sheep serum are removed and passed through gel columns laced with the substance of interest. The antibodies with high affinities for the substance are retained and isolated. Further modifications can be made to the proteins to lessen the chances of adverse reaction [18]. The protein fragments, also known as Fab, are then injected into a patient intravenously to bind the toxin and reverse or reduce toxicity. The advantage of this therapy is the extremely high binding affinities achieved, while the main disadvantages include expensive production and the potential for adverse reactions.

#### 3.2.1. Important Design Parameters

The two primary design criteria for Fab are high binding affinity for the toxin, which should be an inherent property if the Fab are produced correctly, and a low occurrence of adverse reactions. The latter is much more difficult to achieve. One method for reducing toxicity is to isolate the smallest possible antibody fragments so that all extraneous protein regions not useful for binding but capable of causing toxicity are removed. This can be done in a variety of ways, one of which includes protein cleavage with papain [68]. Clinical results have shown that such alterations make Fab treatments reasonably safe. Pharmacokinetic issues are not a concern with Fab, as they are able to cross capillaries and enter the interstitial space [18].

#### 3.2.2. Current and Emerging Fab Therapies

One of the first uses for Fab was for digoxin toxicity [67,68,69]. Lloyd and Smith published an intriguing study in 1978 involving dogs and digoxin [69]. Dogs were treated with non-specific immunoglobulin (IgG), digoxin specific IgG, and digoxin specific Fab. While 0/8 dogs treated with non-specific IgG died, 10/11 dogs given digoxin specific IgG survived, and 6/6 dogs treated with the digoxin specific Fab lived and recovered significantly faster than the other dogs. This study paved the way for the development of Digibind^®^ and DigiFab^®^, two commercial products aimed at digoxin detoxification. Although reviews of clinical results suggest that 91% of treatments are effective, and show lower statistical associations with mortality for treated patients [70], some geographical regions have less than adequate supplies of digoxin binding Fab for treatment [71].

**Figure 10:**
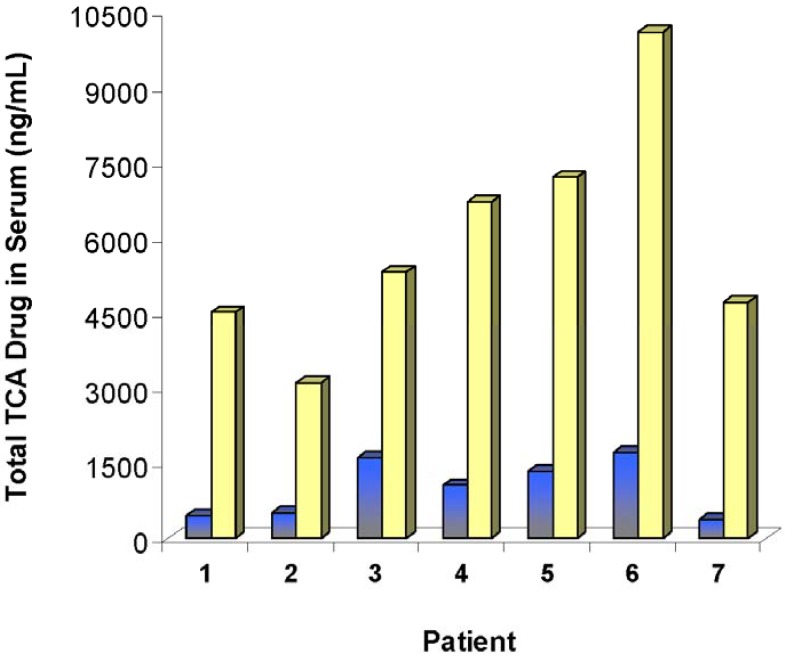
Total serum levels of various tricyclic antidepressant drugs in human patients experiencing drug overdose before treatment (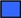) and after a third dose of drug specific antibody fragments (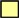) [76].

Another Fab has recently been developed for TCA toxicity [72,73,74,75,76]. The concept was first proven in rats. Treated rats suffering TCA toxicity had longer survival times and higher lethal TCA doses than untreated rats [72]. TCA concentrations in rat serum were also increased ten fold in some cases [73]. More importantly, studies done in animal models were followed up with a small human trial [76]. Patients admitted to the hospital with mild but not life threatening TCA toxicity were treated with TCA specific Fab as a proof of concept. Figure 10 shows that the total amount of TCA in the serum increased dramatically after Fab dosing. Heart rate recoveries were also observed, and no major adverse reactions occurred.

So far, the development of drug-specific Fab has been discussed, but Fab can also be useful for natural toxins. Dart *et al.* isolated Fab capable of binding to crotaline snake venom [77]. The snake subfamily known as crotalinae includes some of the most deadly snakes in the United States, such as the rattlesnake, water moccasin, and copperhead. Although an antivenin product isolated from horses is available [78], it causes severe reactions in 20% to 25% of patients and long term toxicity in 50% to 75% of patients [78]. Following the isolation of the venom specific Fab, Dart *et al.* also conducted a small clinical trial where 31 patients were treated for crotaline snake bite with Fab. 31/31 patients survived and improved. The authors concluded that the Fab treatments were successful, and that multiple Fab injections would be the best treatment option for future patients.

### 3.3. Indirect Treatment Methods

Rather than binding to or acting on toxins directly, indirect detoxification treatments are targeted at reversing or preventing the effects of toxins by interacting with the body. In this way, they closely resemble traditional low molecular weight drugs. Unlike many of the treatments discussed in 3.1 and 3.2, indirect methods have been used clinically for many years. New methods are still emerging, however, such as macroemulsions for local anesthetic reactions. The advantage of indirect treatments is their simplicity. Most lack the complex structures and design constraints of colloids, particles, and antibody fragments, and are therefore less complicated to produce and/or administer. On the other hand, their lack of specificity for a particular toxin often necessitates large doses, which can further injure the patient. This lack of specificity may also render them ineffective as a sole method of treatment when toxin concentrations are very high.

#### 3.3.1. Important Design Parameters

Since indirect treatments act in a variety of ways, the key design parameters vary. Many indirect methods act as antagonists, occupying receptors and preventing toxins from interacting with those receptors and inducing pharmacologic responses. In such cases, high binding affinities between the receptors and the antagonists result in low binding by the toxin. Several antagonists are capable of competing with multiple toxins with similar structures for the same receptor. The toxin structure with the highest degree of receptor binding results in the lowest level of efficacy for the antagonist, and vice versa. Other indirect methods oppose the pharmacologic response induced by the toxin. This opposition may take place at the extracellular or intracellular levels, as well as systemically in the blood compartment. The toxic mode opposed by the treatment dictates what aspect of the treatment must be optimized for sufficient detoxification.

Pharmacokinetics are an important consideration for indirect methods. They are often cleared by hepatic metabolism like drug molecules. Moreover, their degrees of lipophilicity and hydrophilicity determine how fast and to what extent they are taken up by tissues. Pharmacokinetics also play a role in dosing, as rapidly cleared substances have short durations of action and require different methods of dosing. Lastly, the biocompatibility of the indirect treatment method is vital. As mentioned above, indirect treatment methods can require large doses for efficacy. As such, over compensation resulting in toxicity is a concern.

#### 3.3.2. Current and Emerging Indirect Treatment Methods

A myriad of indirect treatment methods have been used over many centuries. While all will not be discussed in this article, several methods that act on the same toxins as the colloids and fragments previously discussed will be covered. This will hopefully provide the reader with a contrasting view of direct and indirect treatment methods for similar substances.

TCA’s were addressed at length during the review of liposomes and Fab. An older method of treating TCA toxicity is the use of sodium bicarbonate (NaHCO_3_) [3,5]. NaHCO_3_ has several modes of action, though the dominant mode is currently up for debate [3,5]. First, intravenous NaHCO_3_ increases the pH of the blood in overdosed patients. The increased pH value results in higher binding affinities between serum proteins and TCA’s, which leads to lower free TCA concentrations [3,5]. However, this mode is highly questionable, as the extent of redistribution following pH changes has not been clinically examined. More than likely, the more important effect is the increased Na concentration near the cell membrane, leading to faster cell membrane depolarization and subsequently faster Na channel recovery [5]. Na channel blockage by TCA’s is a major source of cardiac toxicity.

A less traditional method of treating both BUP and TCA toxicity are macroemulsions. Like microemulsions, macroemulsions are oil droplets stabilized by surfactants or surfactant-like molecules. Unlike microemulsions, macroemulsions are large structures, with each oil droplet being microns in size. Macroemulsions have been used for sometime in hospital and medical care settings as nutrition supplements (intravenous dosing). With a composition consisting of mostly soybean oil and a small amount of phospholipids, they provide essential lipids to diseased patients unable to ingest sufficient quantities orally. While macroemulsions are included within the realm of indirect treatment methods in this review, they could have multiple modes of action [14]. The indirect method seemingly best supported [14] is their ability to overcome reduced adenosine triphosphate (ATP) production by the mitochondria imposed by local anesthetics, the first class of drugs for which they were employed. Increased nitric oxide production has also been mentioned, although to a lesser extent [79]. Finally, they may also induce redistribution, as liposomes and Fab do. Evidence for redistribution by macroemulsions was recently demonstrated by their increased treatment efficacy for more lipophilic compounds [80,81]. More than likely, they are effective at treating toxicity by some combination of the aforementioned methods. Regardless, it is clear that macroemulsions have successfully treated many patients from local anesthetic reactions, in addition to other drugs as well.

Macroemulsions were first investigated as a detoxification therapy by Weinberg *et al.* in the mid to late 1990’s [79]. They showed *in vitro* BUP binding of 75.3% at a macroemulsion concentration of 15% [79]. Note the high concentrations tested and how they compare with liposome concentrations from section 3.1. Their initial studies involved rats, where lethal doses of BUP were five times higher with macroemulsion treatment compared to the control group. Following their rat studies, Weinberg *et al.* continued their work by treating dogs with macroemulsions 10 min after BUP induced toxicity [82]. Amazingly, all treated dogs survived, while all untreated dogs did not. They compared the myocardial tissue oxygen pressure in dogs with and without treatment to show quantitative improvement with emulsions [82]. Since their early work, Dr. Weinberg has established a website for discussing and reporting cases where macroemulsions successfully treated adverse reactions to drugs (www.lipidrescue.org), and many cases have been published [12,13,14,83,84,85]. Most cases involve an immediate adverse reaction to a local anesthetic during surgery, and macroemulsions are typically dosed intravenously within 15 minutes once symptoms fail to subside. A recent review describes the extension of macroemulsions to other drug classes as well [86].

Another group of indirect treatment methods commonly used for detoxification therapy is receptor antagonists. Receptor antagonists directly compete with toxins for access to the binding sites responsible for inducing toxicity. One example of a receptor antagonist is naloxone [22]. Naloxone is an opioid receptor antagonists capable of competing with opioids such as heroin and morphine for mu (µ), kappa (κ), and delta (δ) receptors [22]. Naloxone has been used to effectively treat opioid toxicity since its synthesis in 1960. Opioid antagonists such as naloxone are also used to reduce opioid dependency in drug abusers. Naloxone has a poor bioavailability when taken orally and a rapid onset of action after intravenous administration. Most opioid antagonists produce minimal negative side effects.

Like naloxone, atropine is also a receptor antagonist [87]. Atropine competes with substances for access to muscarinic receptors. Atropine has long been the treatment of choice for exposures to organic phosphorous compounds such as pesticides and chemical warfare agents. When such agents prevent acetylcholinesterase from breaking down acetylcholine, excess acetylcholine begins acting on muscarinic receptors. Acetylcholine normally gets broken down immediately following its release from axons, and when this does not occur, acetylcholine continually acts on the muscarinic receptors to induce severe toxicity. Although atropine is largely well tolerated, other treatment methods such as pralidoxime (2-PAM) are typically given concurrently to increase efficacy [87].

## 4. Conclusions

Based on the information presented here and elsewhere, it is clear that methods for rapid detoxification treatment in critical care situations are necessary within the realms of prescription drugs, drugs of abuse, and exposures to environmental toxins and chemicals. Several new types of detoxification treatment methods continue to be developed and improved upon, including colloids and particles such as liposomes, antibody fragments, and macroemulsions. While the progress made in this field is substantial, there is clearly a need for more progressive research. In particular, more controlled clinical trials are necessary to conclusively show the safety and effectiveness of the treatments. When combined with traditional treatment methods such as sodium bicarbonate and naloxone, these new critical care treatments have the potential to save lives, reduce recovery times, and give physicians new and improved tools for treating patients.
